# Growth Kinetics, Microstructure Evolution, and Some Mechanical Properties of Boride Layers Produced on X165CrV12 Tool Steel

**DOI:** 10.3390/ma16010026

**Published:** 2022-12-21

**Authors:** Natalia Makuch, Michał Kulka, Mourad Keddam, Adam Piasecki

**Affiliations:** 1 Institute of Materials Science and Engineering, Poznan University of Technology, Pl. M.Sklodowskiej-Curie 5, 60-965 Poznan, Poland; 2 Laboratoire de Technologie des Matériaux, Faculté de Génie Mécanique et Génie des Procédés, USTHB, B.P N°32, El-Alia, Bab-Ezzouar, Algiers 16111, Algeria

**Keywords:** powder-pack boriding, growth kinetics of borided layers, hardness, nanomechanical properties, wear resistance, tool steel

## Abstract

The powder-pack boriding technique with an open retort was used to form borided layers on X165CrV12 tool steel. The process was carried out at 1123, 1173, and 1223 K for 3, 6, and 9 h. As a result of boriding the high-chromium substrate, the produced layers consisted of three zones: an outer FeB layer, an inner Fe_2_B layer, and a transition zone, below which the substrate material was present. Depending on the applied parameters of boriding, the total thickness of the borided layers ranged from 12.45 to 78.76 µm. The increased temperature, as well as longer duration, was accompanied by an increase in the thickness of the FeB zone and the total layer thickness. The integral diffusion model was utilized to kinetically describe the time evolution of the thickness of the FeB and (FeB + Fe_2_B) layers grown on the surface of powder-pack borided X165CrV12 steel. The activation energy of boron for the FeB phase was lower than that for the Fe_2_B phase. This suggested that the FeB phase could be formed before the Fe_2_B phase appeared in the microstructure. The high chromium concentration in X165CrV12 steel led to the formation of chromium borides in the borided layer, which increased the hardness (21.88 ± 1.35 GPa for FeB zone, 17.45 ± 1.20 GPa for Fe_2_B zone) and Young’s modulus (386.27 ± 27.04 GPa for FeB zone, 339.75 ± 17.44 GPa for Fe_2_B zone). The presence of the transition zone resulted from the accumulation of chromium and carbon atoms at the interface between the tips of Fe_2_B needles and the substrate material. The presence of hard iron and chromium borides provided significant improvement in the wear resistance of X165CrV12 steel. The powder-pack borided steel was characterized by a four times lower mass wear intensity factor and nine times lower ratio of mass loss to the length or wear path compared to the non-borided material.

## 1. Introduction

Boriding is a thermochemical treatment that is well-known as an effective technique to improve the hardness and wear resistance of non-ferrous as well as ferrous alloys, including different types of steels. The parameters of the process (temperature and time) play key roles in constituting the chemical and phase composition as well as the properties of the steel after boriding. The chemical composition of the treated steel is the second factor influencing the microstructure and properties of the borided layers. In the case of pure iron [1,2,3,4], as well as low-carbon and low-alloy steels [5,6,7], the produced borided layers are characterized by strong zonation and a needle-like microstructure. Depending on the boriding method and its parameters, the microstructure of such layers can be single-phase, containing only Fe_2_B borides [3,7] or can be composed of two types of iron borides (FeB + Fe_2_B) as a dual-phase layer [1,2,4]. In general, a higher temperature and longer duration of the boriding process results in an increase in the borided layer thickness. The phase composition of the boride layer influences the mechanical properties, e.g., the boron-rich FeB phase is significantly harder than the Fe_2_B phase [4]. The various types of iron borides also have different physical properties [4], such as the coefficient of thermal expansion (23·10^−6^/°C and 7.85·10^−6^/°C for FeB and Fe_2_B, respectively) or thermal conductivity (0.12 W/cm·K for FeB and 0.301 W/cm·K for Fe_2_B). Due to the insolubility of carbon in FeB boride and very low solubility in Fe_2_B boride [8], the carbon atoms are moved in a core direction by following the boron diffusion front during boriding. This phenomenon is the reason for the less needle-like morphology of iron borides, as well as the reduction in layer thickness [9,10]. Alloying elements in the steel also reduces the diffusion of boron atoms in a core direction. Therefore, the borided layers produced on high-alloy steels are characterized by lower thickness and flat morphology between the bottom of the iron borides and the substrate material [11,12]. Cr is an alloying element that ensures corrosion resistance in stainless steels and is widely used in tool steels to improve their wear resistance due to the formation of hard carbides. Obviously, the presence of chromium in the steel substrate influences the phase composition, morphology, and properties of the borided layers [13,14,15,16,17]. Cr, as a transition metal, dissolves in iron borides, causing the borided layer produced on high-chromium steel to be predominantly composed of complex phases (Fe,Cr)B (rhombic) and (Fe,Cr)_2_B (tetragonal) [15,17]. Because of the formation of chromium borides, this element also significantly influences the mechanical properties of the borided layers produced on high-chromium steels.

The comparison of boride layers formed on steels differing in chromium content indicated that chromium reduced the thickness of the layer [18]. The thickness of the borided layers was in the range of 8–58 µm for AISI H13 steel (5.3 wt.% of Cr) and 4–42 µm for AISI 304 steel (18.3 wt.% of Cr), depending on the boriding parameters. The produced layers also differed in their mechanical properties. The average hardness of the layer produced on AISI H13 was 1860 HV, whereas the higher chromium content in AISI 304 steel provided a higher hardness of 2150 HV. Simultaneously, the increased chromium concentration in AISI 304 steel resulted in a reduction in the fracture toughness of the boride layer compared to that produced on the substrate with a lower chromium content [18]. Additionally, in the case of tool steels such as AISI M2 steel [19], the morphology, thickness, and properties of the borided layer result from its chemical composition. Alloying elements (Cr, Mo, V, Ni and W) dissolve in iron borides and are partially segregated at the interface between the layer and substrate. Therefore, a smoother morphology is characteristic of the produced interfaces in comparison to the needle-like microstructure of borided layers in low-alloy steels. The presence of chromium borides is the reason for the increased hardness and reduced fracture toughness of the produced layers [19].

In the present study, boride layers were produced on X165CrV12 tool steel using the open retort powder-pack boriding method. The steel was characterized by high carbon and chromium concentrations, the presence of which hindered boron diffusion. The samples were borided using different process parameters: temperatures of 1123, 1173, and 1223 K and times of 3, 6, and 9 h. The growth kinetics of the boride layers and boron activation energies required for their formation were analyzed based on the integral diffusion model. The influence of boriding parameters on the thickness and microstructure evolution of the borided layers is discussed. The effect of boride layer formation on the hardness, nanomechanical properties, and wear resistance of X165CrV12 tool steel was studied.

## 2. Material and Methods

### 2.1. Material

The substrate material used for this study was X165CrV12 cold work tool steel according to the DIN (Deutsches Institut für Normung) standard. The nominal composition is shown in Table 1. This steel contained alloying elements such as Cr and V, which, increased its wear resistance due to the formation of hard carbides. Moreover, the high carbon concentration, as well as the presence of alloying elements (Cr, Si, Mn, V), improved the hardenability of this steel. The samples were in the shape of plates 13 mm × 13 mm × 6.5 mm.

### 2.2. Powder-Pack Boriding

**Figure 1:**
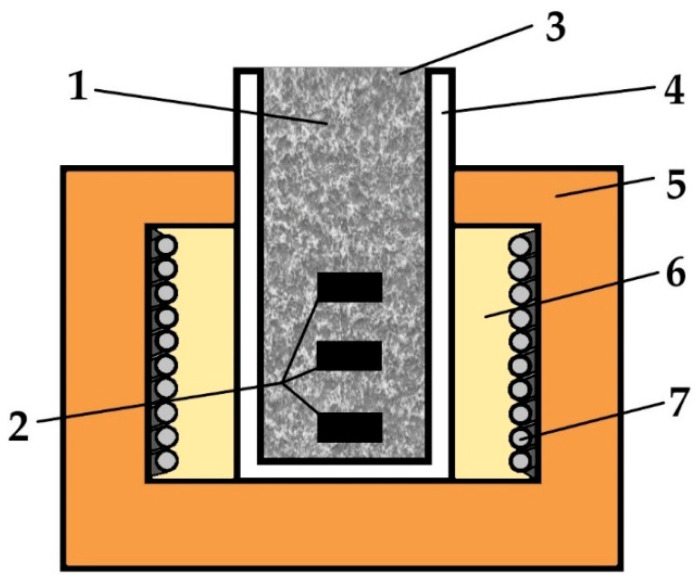
Scheme of device used for powder-pack boriding with an open retort: 1—powder mixture, 2—treated samples, 3—the open upper part of the retort, 4—tube-shaped retort with a closed bottom, 5—electric resistance furnace, 6—furnace chamber, 7—heating elements.

Before thermochemical treatment, the samples were washed with distilled water, degreased with acetone, and rinsed with alcohol. The boriding was carried out in a powder mixture containing 50 wt.% B_4_C as the boron source, 0.5 wt.% AlF_3_ as an activator, and 49.5 wt.% Al_2_O_3_ as a diluent. The technique used in the present study for boriding was powder-pack boriding with an open retort. This is a unique technique carried out using the device shown in Figure 1. The tube-shaped retort, made of stainless steel with a closed bottom, was filled with the samples and the boriding medium (powder mixture) without an additional seal. The prepared retort was placed in the chamber of an electric resistance furnace so that its upper part extended outside the furnace (Figure 1). As a result, the powder mixture in the upper part of the retort (above the furnace) became a natural seal. Moreover, the temperature of the boriding powder in the upper part of the retort was so low that this mixture was protected against oxidation. Simultaneously, the gases from the bottom part of the retort were subjected to re-sublimation in the cold upper part. Hence, these gases did not escape beyond the retort [20].

The samples were borided using different process parameters: temperatures of 1123, 1173, and 1223 K and durations of 3, 6, and 9 h. The position of the sample in the powder mixture was chosen so that its temperature was consistent with that assumed. The temperature was verified using a thermocouple. In all cases, boriding was carried out at one fixed temperature for one specific period of time.

### 2.3. Microstructure and Property Characterization

The phases in the borided layers were identified by X-ray diffraction (XRD) analysis using a PANalytical EMPYREAN computer-controlled X-ray diffractometer (Malvern Panalytical Ltd., Poznań, Poland). CuKα radiation (λ = 0.154 nm) with an angle of 2θ in the range from 30° to 90° was used for this study.

For the metallographic examination, the borided samples were cut and the cross-sections were hot-mounted in a conductive resin. The specimens were then ground using SiC abrasive paper and polished with 0.05 µm aluminum oxide paste. A hot-etching technique was used to reveal details in the microstructure of the borided layers. A special reagent, consisting of potassium ferrocyanide, potassium ferricyanide, and potassium hydroxide, was used. The microstructural observations were carried out using the LAB-40 optical microscope (OM; OPTA-TECH, Poznan, Poland). Microstructural characterization was also performed using a Mira 3 scanning electron microscope (SEM; TESCAN, Poznan, Poland) equipped with an energy dispersive spectrometer (EDS). The concentrations of Fe, Cr, and B were measured in the cross-section of the borided layer. The thickness of the borided layer was calculated as an average value from approximately 100 measurements carried out at different locations in the cross-section of the metallographic sample. The main problem during these measurements was the needle-like morphology of the borides. The thickness of the FeB zone and the total layer thickness (FeB + Fe_2_B) were measured using the procedure proposed by Kunst and Schaaber [4]. The measurements were performed using OM images of the borided layers. The thicknesses of the FeB zone and the entire FeB + Fe_2_B layer were measured at constant intervals. This took into account the needle-like morphology of the borides.

The microhardness profile across the boride layer was investigated using a Micromet II hardness tester (Buehler, Poznan, Poland). The Vickers diamond indenter under a load of 50 gf (0.49 N) and a peak-load contact of 15 s was used for the measurements.

The nanomechanical properties were tested for the borided samples prepared at 1223 K for 9 h. For this study, an NHT^3^ nanoindentation tester (Anton Paar, Poznan, Poland) equipped with a Berkovich diamond indenter was used. The mechanical properties were estimated according to Oliver and Pharr’s method [21,22]. Measurements were carried out under a maximum load *F_max_* of 50 mN with loading and unloading rate of 100 mN/min. As a consequence of these measurements, the load-displacement curves, indentation hardness (*H_IT_*), and indentation Young’s modulus (*E_IT_*) were determined. Poisson’s ratio (*v_s_*) is an important parameter required to determine Young’s modulus. The value of the Poisson’s ratio strongly depends on the type of the examined microstructure. In the present study, the nanomechanical properties for the FeB zone, Fe_2_B zone, and the substrate material were investigated. In the case of the outer zone, which contained (Fe,Cr)B borides, an average value (*v_s_* = 0.2265) was calculated based on the Poisson’s ratio of the FeB phase (*v_s_* = 0.25 [23]) and the CrB phase (*v_s_* = 0.203 [24]). For the Fe_2_B phase, a value of 0.25 was taken for the calculations, according to the literature data [23]. The substrate material was characterized by a Poisson’s ratio of 0.285. The selected Berkovich indents performed in each zone were observed using a Mira 3 scanning electron microscope (SEM; TESCAN, Poznan, Poland).

**Figure 2:**
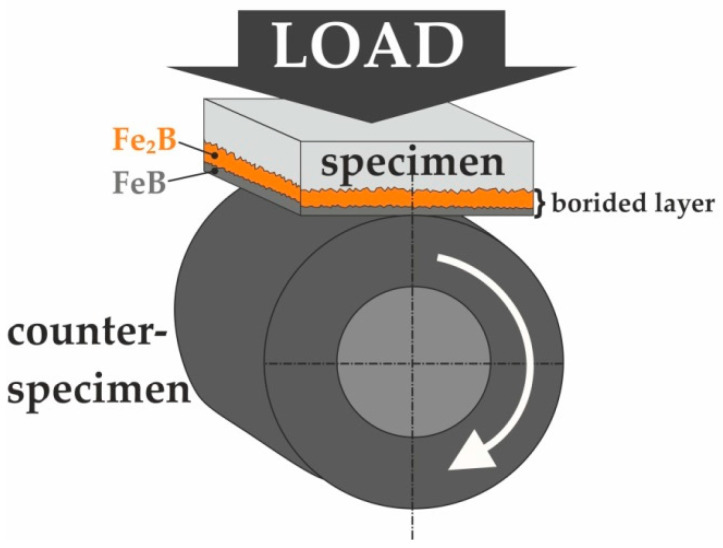
Scheme of the frictional pair during wear tests of powder-pack borided and non-borided X165CrV12 cold work tool steel; load *F* = 49 N, *n* = 250 min^−1^.

The wear resistance tests were performed under the conditions of unlubricated dry friction. The friction pair consisted of an immobile specimen (in the shape of a plate) and a mobile counter-specimen (in a shape of a ring with an external diameter of 20 mm, internal diameter of 12 mm, and height of 12 mm). The counter-specimen was made of quenched and low-tempered 100CrMnSi6-4 bearing steel with a hardness of 64 HRC. The following parameters were used during the tests: load of 5 kgf (49 N), counter-specimen rotation speed (*n*) of 250 min^−1^, duration of 4 h. The scheme for the wear resistance test with the positions of the specimen and counter-specimen is shown in Figure 2. The initial contact area between the specimen and counter-specimen was linear. As the wear time progressed, a friction path was formed in the tested sample. The shape of this friction path represented the shape of the outer cylindrical surface of the counter-specimen with a radius of approximately 10 mm. Therefore, the contact pressure changed during the wear test and was difficult to determine. The tribological properties were investigated for two specimens: non-borided X165CrV12 steel and X165CrV12 steel borided at 1223 K for 9 h. Although the total duration of the test was 4 h, the masses of the specimen and counter-specimen were measured every 30 min of the test. Based on the obtained results, three parameters describing the wear resistance were determined: the mass wear intensity factor (*I_mw_*) of the specimens and counter-specimens, the ratio of mass loss to the length of the wear path (∆*m*/*l*) for the specimens, and the relative mass loss (Δ*m/m_i_*) for the counter-specimens.

The mass wear intensity factor was defined as the mass loss (Δ*m*) per friction surface (*S*) and unit of friction time (*t*). Its value corresponded to the slope of a straight line in the diagram of mass loss per friction surface (∆*m*/*S*) versus friction time (*t*). The values *of I_mw_* were calculated according to the equation:

(1) $$I_{mw}=\frac{\Delta m}{S\cdot t}$$

where ∆*m*—mass loss of the specimen or counter-specimen (mg), *S*—friction surface (cm^2^), *t*—friction time (h).

In the case of specimens, an additional calculated parameter was the ratio of mass loss to the length of the wear path (∆*m*/*l):*

(2a) $$\frac{\Delta m}{l}=\frac{m_{i}-m_{f}}{l}$$

where Δ*m*—mass loss of the specimen (mg), *m_i_*—initial mass of the specimen (mg), *m_f_*—final mass of the specimen (mg), *l*—final length of wear path (mm).

The relative mass loss (Δ*m/m_i_*) of the counter-specimens was defined as a mass loss (Δ*m*) in relation to the initial mass (*m_i_*), according to the equation:

(2b) $$\frac{\Delta m}{m_{i}}=\frac{m_{i}-m_{f}}{m_{i}}$$

where Δ*m*—mass loss of the counter-specimen (mg), *m_i_*—initial mass of the counter-specimen (mg), *m_f_*—final mass of the counter-specimen (mg).

The specimens and counter-specimens were weighed on an AS 60/220.R2 analytical balance (RADWAG, Poznań, Poland) to an accuracy of 0.01 mg. Every 30 min of the test, the friction surface was measured and calculated. The entire cylindrical outer surface of the counter-sample was assumed as its friction surface. Based on the measurements of width and length of the wear path using an optical microscope, the friction surface of the samples was calculated.

## 3. Results and Discussion

### 3.1. Microstructure of Borided Layers

**Figure 3:**
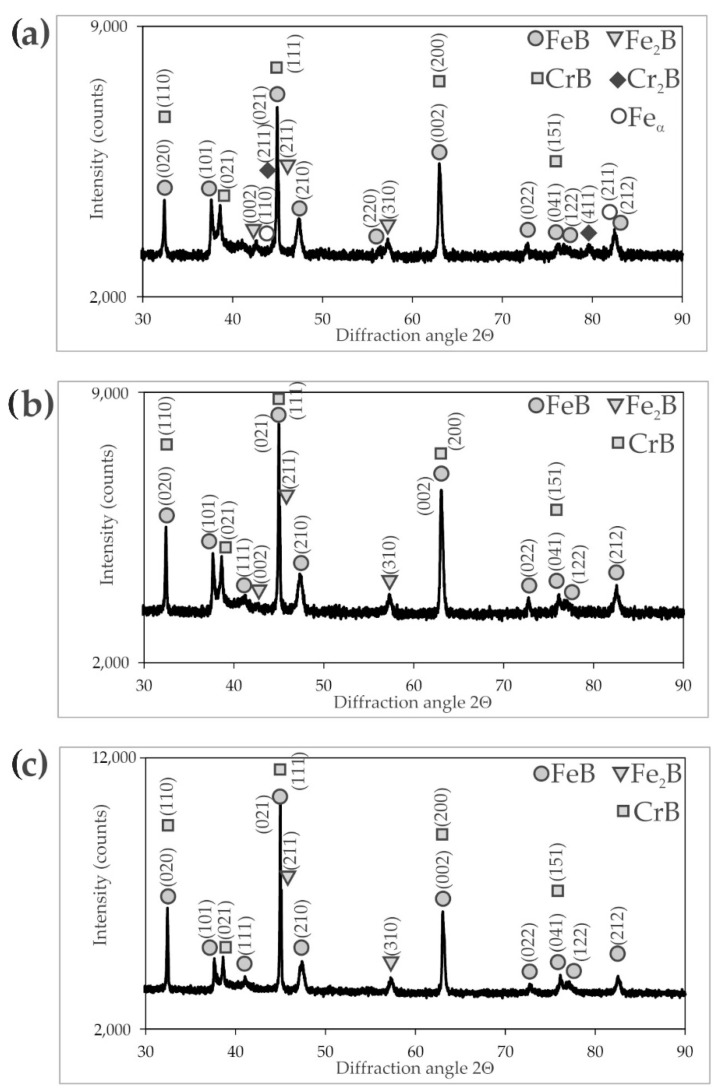
XRD patterns of X165CrV12 steel after powder-pack boriding at 1123 K: (**a**) for 3 h, (**b**) for 6 h, (**c**) for 9 h.

**Figure 4:**
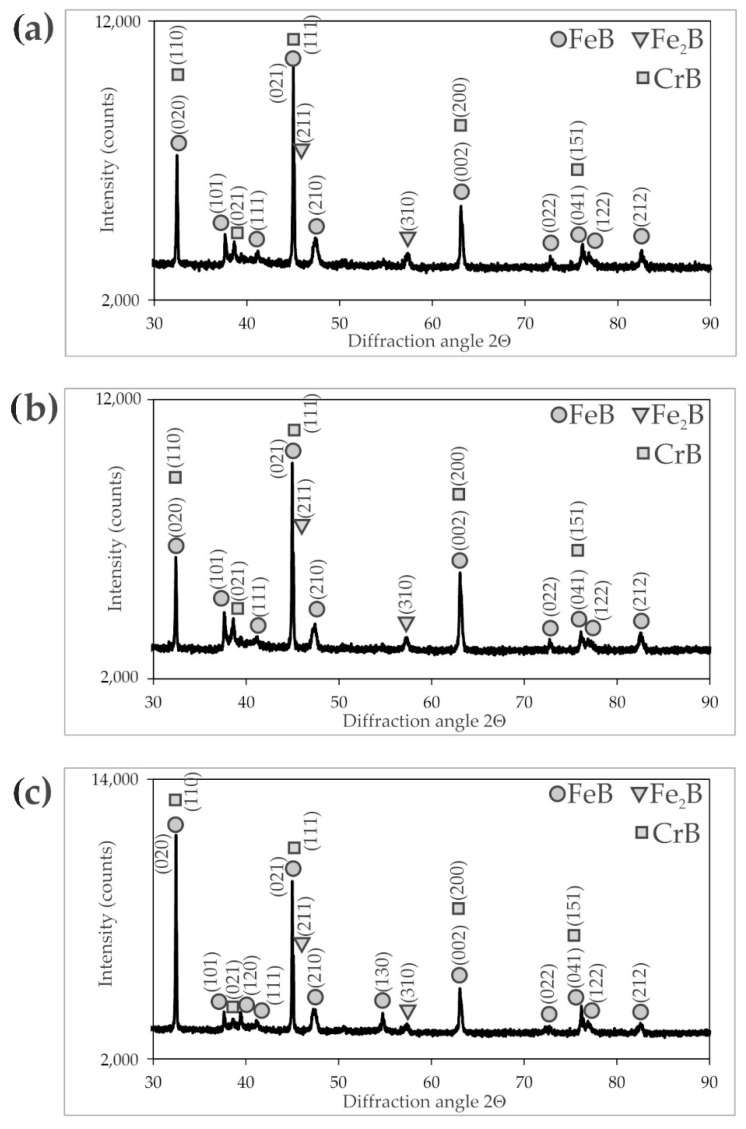
XRD patterns of X165CrV12 steel after powder-pack boriding at 1173 K: (**a**) for 3 h, (**b**) for 6 h, (**c**) for 9 h.

**Figure 5:**
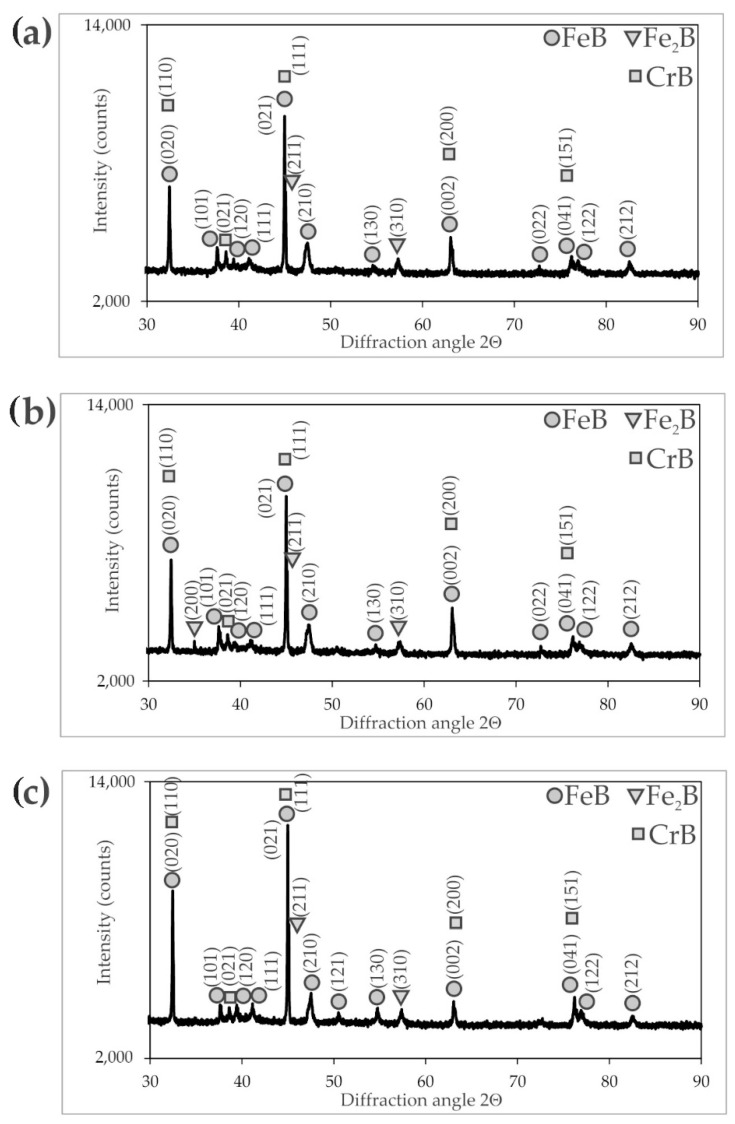
XRD patterns of X165CrV12 steel after powder-pack boriding at 1223 K: (**a**) for 3 h, (**b**) for 6 h, (**c**) for 9 h.

**Figure 6:**
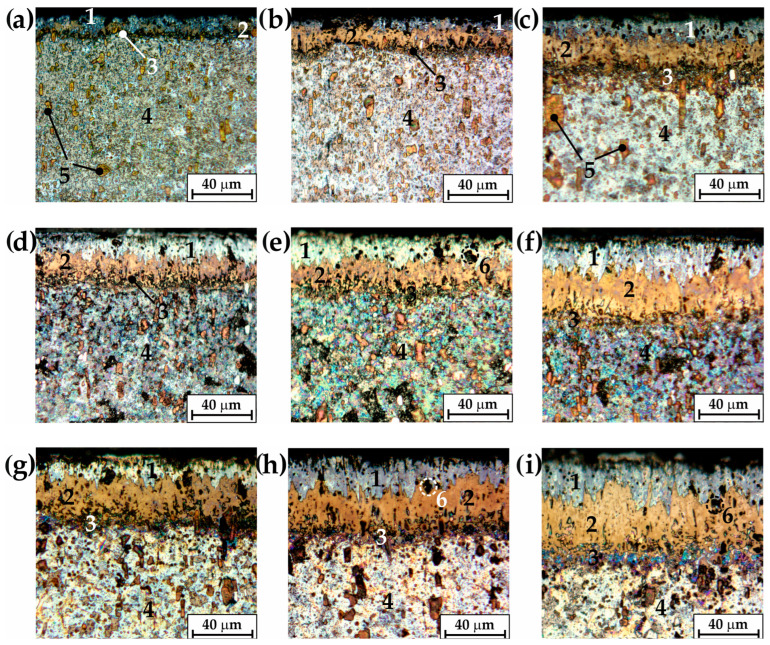
Microstructure of X165CrV12 tool steel after powder-pack boriding using various parameters: (**a**) 1123 K for 3 h, (**b**) 1123 K for 6 h, (**c**) 1123 K for 9 h, (**d**) 1173 K for 3 h, (**e**) 1173 K for 6 h, (**f**) 1173 K for 9 h, (**g**) 1223 K for 3 h, (**h**) 1223 K for 6 h, (**i**) 1223 K for 9 h; 1—FeB zone, 2—Fe_2_B zone, 3—transition zone, 4—substrate material, 5—carbides, 6—pores resulting from the carbides’ removal during the metallographic preparation of the samples.

The X-ray diffraction patterns (Figure 3, Figure 4 and Figure 5) of the borided X165CrV12 steel confirmed the formation of dual-phase layers composed of two types of iron borides: FeB and Fe_2_B. Due to the high concentration of chromium in the substrate material (11–12 wt.%), chromium borides (CrB) were also identified. In the case of the layer produced at the lowest temperature (1123 K) and shortest duration (3 h), the presence of Fe_α_ was detected as well as Cr_2_B borides. The reason for this situation was the relatively low thickness of the borided layer. Hence, the XRD radiation also penetrated the base material. In summary, the microstructure of the powder-pack borided layers consisted of two main zones: the outer FeB zone and the Fe_2_B zone below the first zone. The main phases in these zones were FeB and Fe_2_B borides, respectively. Additionally, CrB borides probably appeared in the outer FeB zone and Cr_2_B borides appeared in the Fe_2_B zone in smaller amount. It is also possible that chromium replaced iron in the complex borides (Fe,Cr)B or (Fe,Cr)_2_B. With increasing boriding temperature and time, the peaks from the FeB and CrB phases became more dominant in the XRD patterns. This was due to the increasing thickness of the outer FeB zone, as shown in Figure 6.

All of the produced layers consisted of the three zones (Figure 6): the outer FeB layer (1), the Fe_2_B boride zone below the first zone (2) and the transition zone (3). Beneath the borided layer, the substrate material (4) was visible. Chromium high-carbon steel was used as the substrate material. Therefore, alloyed carbides (5) were observed in the substrate, as indicated in Figure 6a,c. The average total thickness of the borided layer increased with increasing temperature and duration of the boriding process. Obviously, the parameters of the boriding process also influenced the thickness of the outer FeB layer. In some areas (Figure 6e,h,i), the characteristic pores (6) were visible. These pores were formed as a consequence of carbide removal during the metallographic preparation of the samples. Characteristic of the microstructure of X165CrV12 borided steel was a very irregular boundary between Fe_2_B borides and the substrate material. Carbides likely occurred in this zone, so this zone was called the transition zone to distinguish it from the other zones.

**Figure 7:**
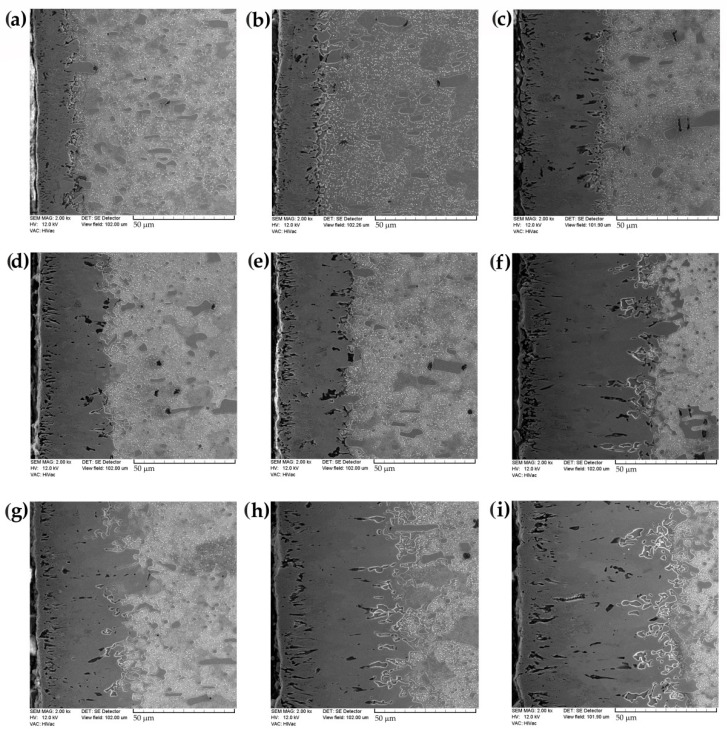
SEM microstructure of X165CrV12 tool steel after powder-pack boriding using various parameters: (**a**) 1123 K for 3 h, (**b**) 1123 K for 6 h, (**c**) 1123 K for 9 h, (**d**) 1173 K for 3 h, (**e**) 1173 K for 6 h, (**f**) 1173 K for 9 h, (**g**) 1223 K for 3 h, (**h**) 1223 K for 6 h, (**i**) 1223 K for 9 h.

The OM images of the microstructure provided basic information about the zones identified in the borided X165CrV12 steel. They were used to measure the thickness of the outer FeB zone as well as the total layer thickness. Representative SEM images are shown in Figure 7. The layers, produced at 1123 K, were characterized by an average thickness from 12.45 to 38.53 µm with increasing processing time from 3 h to 9 h (Figure 7a–c). A further increase in the boriding temperature resulted in the formation of a layer with a thickness ranging from 33.03 to 66.96 µm, depending on the duration of the process (Figure 7d–f). The highest temperature of boriding (Figure 7g–i) ensured the highest thickness of layer, with 45.07, 59.62, and 78.76 µm produced at processing times of 3, 6, and 9 h, respectively.

**Figure 8:**
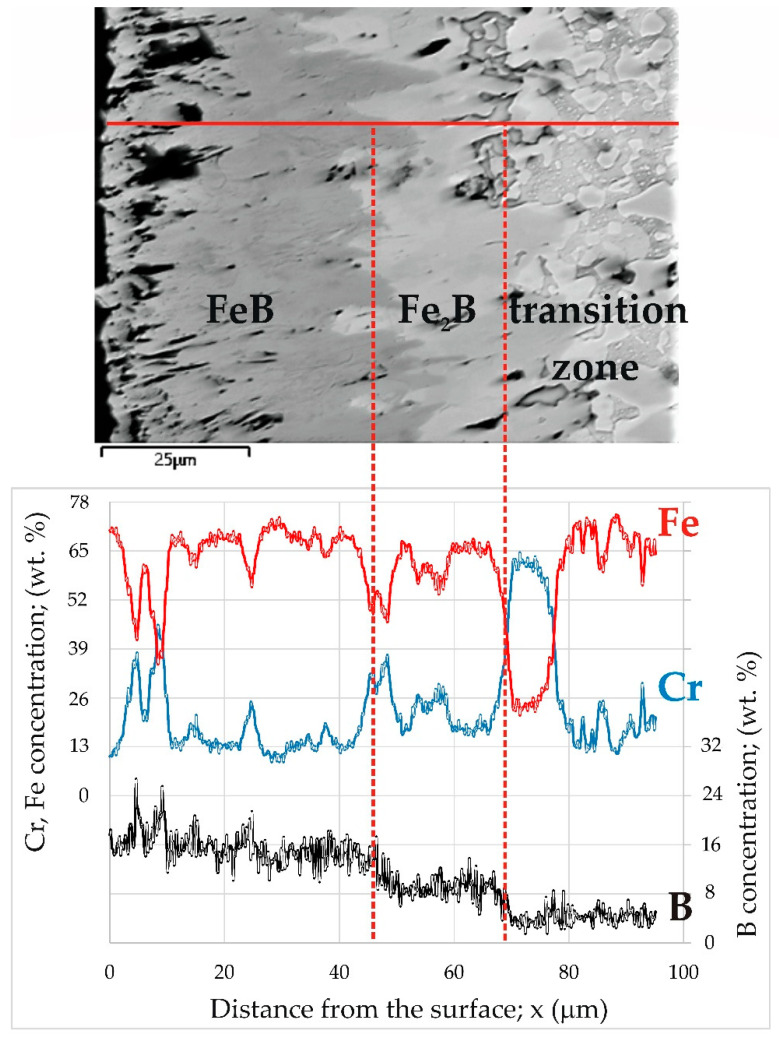
BSE image and results of line X-ray microanalysis performed for X165CrV12 tool steel after powder-pack boriding at the temperature of 1223 K for 9 h.

**Figure 9:**
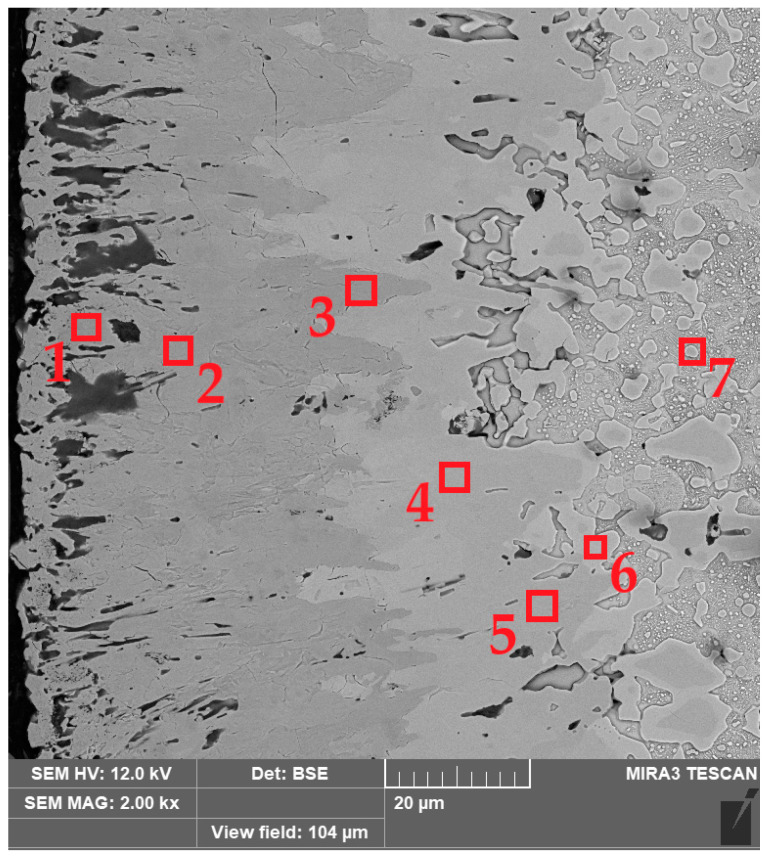
BSE image of microstructure of X165CrV12 tool steel after powder-pack boriding at the temperature of 1173 K for 9 h with marked areas examined by EDS microanalysis.

The identification of phases in the cross-sections of the borided layers required the use of EDS X-ray microanalysis (Figure 8). This investigation was performed for the sample produced at of 1223 K for 9 h. This sample was selected due to the highest thickness of the boride layer, which enabled the differences in chemical composition to be observed most clearly. For the other boriding parameters, the concentration profiles of the elements would be similar, but less clear due to thinner layers. First, the distributions of iron, chromium, and boron were measured along a line through all zones (Figure 8). The distribution of boron in the cross-section of the borided X165CrV12 steel strongly depended on the distance from the surface. In general, with increasing distance from the surface, the concentration of boron decreased. The highest values of boron content were measured in the outer FeB zone (approximately 16.7 wt.%). The obtained average value of boron concentration was consistent with the equilibrium content of boron in the FeB phase (16.22 wt.%). However, in some areas, especially near the top surface, the local maxima (in the distance from the surface of 5, 9, 15, and 25 µm) of the boron concentration was observed. Simultaneously, the local maxima of the chromium concentration and the local minima of the iron concentration were observed in these areas. This indicated the presence of chromium borides in the outer zone, which had a higher boron content than iron borides. The presence of the CrB phase was confirmed by the XRD phase analysis (Figure 5i). The inner Fe_2_B zone was characterized by the lowest concentration of boron (approximately 9.2 wt.%). This value was comparable to the equilibrium content of boron in the Fe_2_B phase (8.83 wt.%). A further increase in the distance from the surface resulted in a reduced boron concentration (Figure 8). This was accompanied by a change in the microstructure from the iron boride layer to the transition zone. The interface between the tips of Fe_2_B needles and the substrate material was characterized by increased chromium concentration. Simultaneously, the iron and boron concentrations were significantly reduced in this area. This indicated the presence of chromium carbides in this zone. To confirm this, EDS X-ray microanalysis was performed in the selected areas in the microstructure of the borided layer produced (Figure 9).

EDS X-ray microanalysis was performed in seven selected areas of the borided layer produced at 1173 K for 9 h (Figure 9). The concentrations of chromium, iron, and boron were measured, and the results are presented in Table 2. It was confirmed that an increased chromium concentration (33.7 wt.%) was measured in area 1 (near the top surface). Simultaneously, this region was characterized by a high boron (18.8 wt.%) and iron (47.5 wt.%) concentrations. This composition suggested the presence of a MeB type boride in this region, where Me = Fe, Cr. It is well known that both FeB and CrB borides crystallize in the orthorhombic structure and show a tendency to give rise to mixed or complex borides [8,25,26]. The studied areas 2 and 3 were also found in FeB needles, but their chemical composition indicated a high iron content and rather low chromium content. Simultaneously, these areas were characterized by the boron content typical of the FeB phase. In the case of areas 4 and 5, the measured boron concentration confirmed the presence of Fe_2_B borides in the layer. Notably, an increased chromium concentration was obtained in area 4 as a result of the accumulation of chromium atoms and its good solubility in Fe_2_B boride. This situation suggested the formation of the alloyed (Fe,Cr)_2_B phase [17], although the phase analysis using XRD (Figure 3, Figure 4 and Figure 5) did not confirm its presence. In the transition zone (areas 6 and 7), a significantly reduced boron concentration was obtained. The area marked as 6 (Figure 9) contained a higher concentration of chromium (15.3 wt.%) compared to the nominal content of this element in X165CrV12 steel (11–12 wt.%). It was previous reported [13,16] that during boriding of high-chromium steels, chromium concentrated at the interface between Fe_2_B needles and the matrix. In the case of high-carbon steels, during saturation by boron, carbon atoms are pushed towards the substrate material. Due to the insolubility of carbon in FeB and its very low solubility in the Fe_2_B phase, carbon atoms are pushed towards the base material in high-carbon steels during saturation with boron [8]. Therefore, at the interface between the Fe_2_B zone and the substrate material, there were more favorable conditions to form substitute carbides of the Me_3_C type, in which the iron atoms were partially replaced by chromium atoms. At a higher depth of 95 µm from the top surface (area 7), a characteristic chromium concentration (11.6 wt.%) was obtained as in the typical nominal composition of X165CrV12 steel (Table 1). It was assumed that area 7 corresponded to an alloyed cementite (Fe,Cr)_3_C typical of high-chromium tool steel.

**Figure 10:**
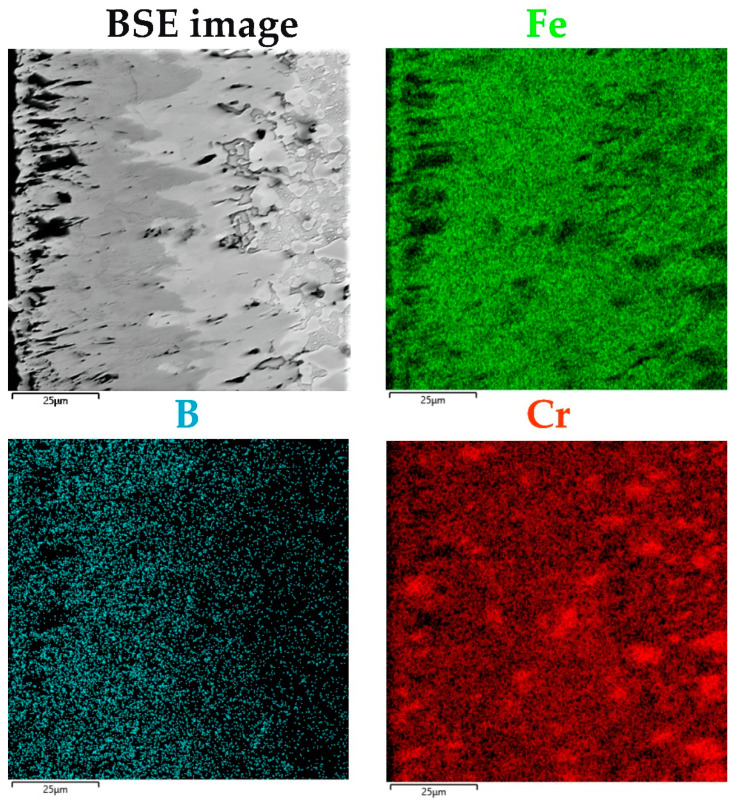
BSE images and results of EDS mapping performed for X165CrV12 tool steel after powder-pack boriding at the temperature of 1223 K for 9 h.

The preferential accumulation of chromium below the iron borides layer was confirmed by EDS mapping (Figure 10) of elements characteristic of borided high-chromium steel (Cr, Fe, B). It was clearly visible that in some areas at the interface between the Fe_2_B zone and the substrate material, the increased concentration of chromium was accompanied by a reduced iron concentration. The etching of the samples, used in the present study, was aimed at revealing of FeB and Fe_2_B zones of different colors, rather than revealing the structure of the substrate. Hence, it was difficult to observe the microstructure of the substrate material. However, X165CrV12 steel belongs to ledeburitic steels, i.e., after slow cooling after the boriding process, it has a structure consisting of fine pearlite, secondary carbides, and ledeburite—a transformed eutectic mixture—which includes fine pearlite, as well as primary and secondary carbides. It was assumed that in the areas below the Fe_2_B zone, such a microstructure occurred with high-chromium carbides.

### 3.2. Diffusion Model

#### 3.2.1. The Integral Diffusion Model

**Figure 11:**
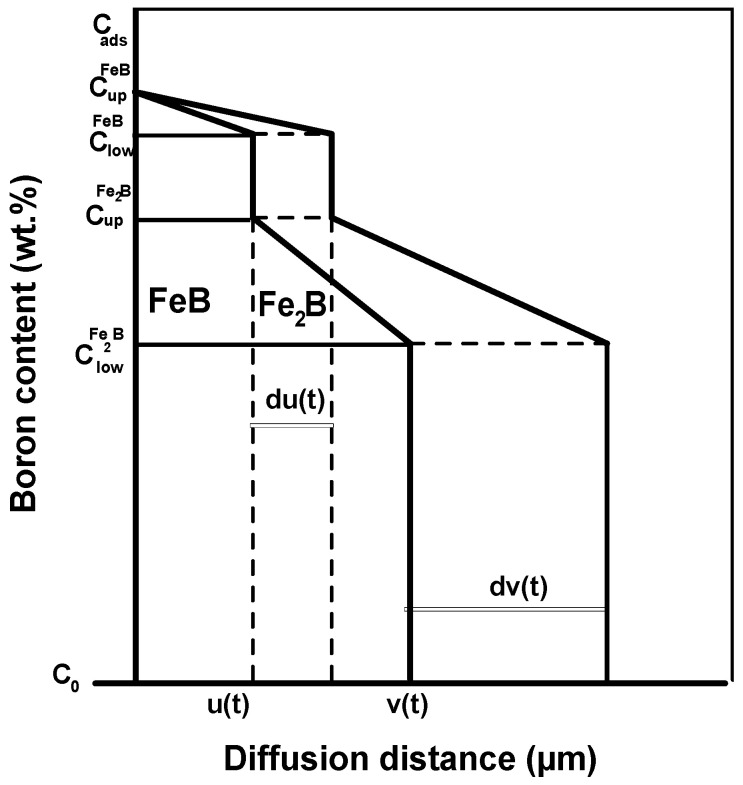
Graphical description of boron concentration profiles along the bilayer (FeB + Fe_2_B).

A recent version of this mathematical model [27] was utilized to kinetically describe the time evolution of the thickness of the layers on the surface of powder-pack borided X165CrV12 steel. The diffusion phenomenon occurred in a semi-infinite medium saturated with boron atoms. Figure 11 graphically illustrates the generated boron concentration profiles inside the FeB and Fe_2_B layers without the incubation times.

The boron concentrations at the growing interfaces were kept constant no matter the treatment time and process temperature. The upper and lower limits in FeB were $C_{up}^{FeB}$ (16.40 wt.%B) and $C_{low}^{FeB}$ (16.23 wt.%B), respectively. For the Fe_2_B phase, the maximum and minimum boron concentrations were $C_{up}^{Fe_{2}B}$ (9 wt.%B) and $C_{low}^{Fe_{2}B}$ (8.83 wt.%B), respectfully [28]. $C_{ads}$ is the adsorbed boron concentration at the beginning of boronizing process at the steel surface [29]. 

The variable *u*(*t*) designates the first time of the growing interface (FeB/Fe_2_B) and *ν(t)* represents the second interface (Fe_2_B/substrate). The solubility of boron atoms within the matrix was extremely low and had a value of 35·10^−4^ wt.% B) [30].

The time evolution of the FeB layer’s thickness can be expressed by Equation (3):

(3) $$u\left(t\right)=k^{\prime }\sqrt{t}$$

where *k′* refers to the parabolic growth constant relative to the first interface (FeB/Fe_2_B). 

The change in time of the entire boride (FeB + Fe_2_B) layer’s thickness is ruled by Equation (4):

(4) $$v\left(t\right)=k\sqrt{t}$$

where *k* refers to the parabolic growth for the second interface (Fe_2_B/substrate).

The initial and boundary conditions used during the establishment of the integral diffusion model [27] are given by the following:

(5) $$C_{FeB}\left\{x\left(t>0\right)=0\right\}=0;\;C_{Fe_{2}B}\left\{x\left(t>0\right)=0\right\}=0;\;C_{Fe}\left\{x\left(t>0\right)=0\right\}=0$$

(6) $$C_{FeB}\left\{x\left[t=0\right]=0\right\}=C_{up}^{FeB}\;\mathrm{for}\;C_{ads}>C_{low}^{FeB}$$

(7) $$C_{FeB}\left\{x\left[t=0\right]=0\right\}=C_{low}^{FeB}\;\mathrm{for}\;C_{ads}<C_{low}^{FeB}\;\mathrm{and}\;\mathrm{with}\;\mathrm{FeB}\;\mathrm{phase}$$

(8) $$C_{Fe_{2}B}\left\{x\left[t=0\right]=0\right\}=C_{up}^{Fe_{2}B}\;\mathrm{for}\;C_{low}^{Fe_{2}B}<C_{ads}<C_{low}^{FeB}\;\mathrm{and}\;\mathrm{without}\;\mathrm{FeB}\;\mathrm{phase}$$

(9) $$C_{Fe_{2}B}\left\{x\left[t=0\right]=0\right\}=C_{low}^{Fe_{2}B}\;\mathrm{for}\;C_{ads}<C_{low}^{Fe_{2}B}\;\mathrm{and}\;\mathrm{without}\;\mathrm{FeB}\;\mathrm{phase}$$

(10) $$C_{FeB}\left(x\left(t=t\right)=u\right)=C_{low}^{FeB}$$

(11) $$C_{Fe_{2}B}\left(x\left(t=t\right)=u\right)=C_{up}^{Fe_{2}B}$$

(12) $$C_{Fe_{2}B}\left(x\left(t=t\right)=u\right)=C_{low}^{Fe_{2}B}$$

(13) $$C_{Fe}\left(x\left(t=t\right)=v\right)=C_{0}$$

The boron distribution profiles across the FeB and Fe_2_B layers are formulated by Equations (14) and (15) [31]:

(14) $$C_{FeB}\left(x,t\right)=C_{low}^{FeB}+a_{1}\left(t\right)\left(u\left(t\right)-x\right)+b_{1}\left(t\right){\left(u\left(t\right)-x\right)}^{2}\;\mathrm{for}\;0\leq x\leq u$$

(15) $$C_{Fe_{2}B}\left(x,t\right)=C_{low}^{Fe_{2}B}+a_{2}\left(t\right)\left(v\left(t\right)-x\right)+b_{2}\left(t\right){\left(v\left(t\right)-x\right)}^{2}\;\mathrm{for}\;u\leq x\leq v$$

This diffusion problem can be solved using the following system of differential algebraic equations (DAE) provided by Equations (16) to (20).

(16) $$a_{1}\left(t\right)u\left(t\right)+b_{1}\left(t\right)u{\left(t\right)}^{2}=\left(C_{up}^{FeB}-C_{low}^{FeB}\right)$$

(17) $$a_{2}\left(t\right)l\left(t\right)+b_{2}\left(t\right)l{\left(t\right)}^{2}=\left(C_{up}^{Fe_{2}B}-C_{low}^{Fe_{2}B}\right)$$

(18) $$\frac{d}{dt}\left[\frac{u{\left(t\right)}^{2}}{2}a_{1}\left(t\right)+\frac{u{\left(t\right)}^{3}}{3}b_{1}\left(t\right)\right]=2D_{FeB}b_{1}\left(t\right)u\left(t\right)$$

(19) $$2w_{12}\frac{dv\left(t\right)}{dt}+\frac{{\left(v\left(t\right)-u\left(t\right)\right)}^{2}}{2}\frac{da_{2}\left(t\right)}{dt}+\frac{{\left(v\left(t\right)-u\left(t\right)\right)}^{3}}{3}\frac{db_{2}\left(t\right)}{dt}\;=2D_{Fe_{2}B}b_{2}\left(t\right)\left(v\left(t\right)-u\left(t\right)\right)$$

(20) $$\left[a_{1}^{2}\left(t\right)-2w_{1}b_{1}\left(t\right)\right]D_{FeB}=a_{1}\left(t\right)\left[a_{2}\left(t\right)+2b_{2}\left(t\right)\left(v\left(t\right)-u\left(t\right)\right)\right]D_{Fe_{2}B}$$

(21) $$2w_{12}a_{2}\left(t\right)b_{1}\left(t\right)D_{FeB}=a_{1}\left(t\right)\left[a_{2}^{2}\left(t\right)-2w_{2}b_{2}\left(t\right)(v\left(t\right)\right]D_{Fe_{2}B}$$

With $w_{1}=\left[\frac{\left(C_{up}^{FeB}+C_{low}^{FeB}\right)}{2}-C_{up}^{Fe_{2}B}\right],$ $w_{2}=\left[\frac{C_{up}^{Fe_{2}B}+C_{low}^{Fe_{2}B}}{2}-C_{0}\right]$ and $w_{12}=\frac{C_{up}^{Fe_{2}B}-C_{low}^{Fe_{2}B}}{2}$.

By employing a particular solution of this system [27] and also considering Equations (22) and (23):

(22) $$u\left(t\right)=2\varepsilon \sqrt{D_{FeB}t}$$

(23) $$v\left(t\right)=2\eta \sqrt{D_{Fe_{2}B}t}$$

The nonlinear system of equations [27] obtained after making appropriate variable changes allows for the numerical determination of the two dimensionless parameters *ε* and *η* based on the Newton-Raphson algorithm [32]. Afterwards, the boron diffusion coefficients in FeB and Fe_2_B can be assessed from Equations (24) and (25):

(24) $$D_{FeB}={\left(\frac{k^{\prime }}{2\varepsilon }\right)}^{2}$$

(25) $$D_{Fe_{2}B}={\left(\frac{k}{2\eta }\right)}^{2}$$

With:

(26) $$\varepsilon =\sqrt{\frac{\beta_{1}}{\left(\frac{a_{1}}{2}+\frac{\beta_{1}}{3}\right)}}$$

(27) $$\eta =\sqrt{\frac{\beta_{2}k^{2}}{\left[2w_{12}k\left(k-k^{\prime }\right)-\left(\frac{a_{2}}{2}+\frac{2\beta_{2}}{3}\right){\left(k-k^{\prime }\right)}^{2}\right]}}$$

#### 3.2.2. Deduced Results from the İntegral Method

**Figure 12:**
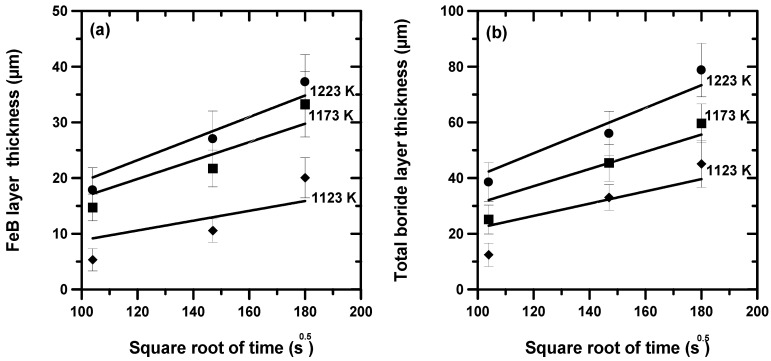
Change in thickness of layers over time for three process temperatures: (**a**) FeB layer, (**b**) (FeB + Fe_2_B).

The experimental results were analyzed to kinetically describe the time variation of the thicknesses of the layers for a given process temperature. Therefore, plots showing the change in the thicknesses of the FeB and (FeB + Fe_2_B) layers versus the square root of time duration were obtained. Figure 12 describes the time dependence of the layers’ thicknesses at increasing process temperatures. The trend of the plotted straight lines demonstrated the parabolic characteristics for the growth kinetics of the borided layers. Table 3 groups the experimental parabolic constants derived from the slopes of the plotted straight lines in Figure 12 for the two growth fronts.

Table 4 gives the estimated boron diffusion coefficients in the two iron borides (FeB and Fe_2_B) calculated with Equations (24) and (25) along with the numerical values of the two dimensionless parameters ε and *η*. Notably, the process temperature had a negligible effect on these two parameters.

**Figure 13:**
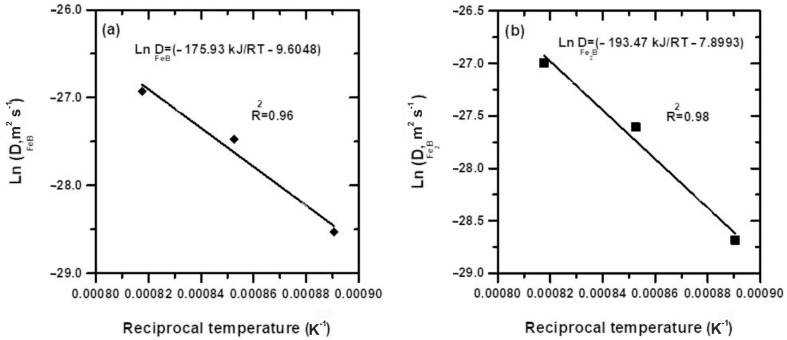
Plots of Arrhenius laws for boron diffusion coefficients in the two borides: (**a**) FeB (**b**) Fe_2_B.

Figure 13 shows the temperature dependencies of the assessed boron diffusion coefficients in the two iron borides FeB and Fe_2_B. Hence, the plots make it easy to deduce the boron activation energies for this type of steel by searching for their numerical values from the slopes of the obtained straight lines. By fitting the calculated values of the boron diffusivities in iron borides using Arrhenius relations, it is possible to derive Equations (28) and (29):

(28) $$D_{FeB}=6.74\cdot 10^{-5}exp\left(\frac{-173.73\frac{\mathrm{kJ}}{\mathrm{mol}}}{RT}\right)\;\;\;m^{2}/s$$

(29) $$D_{Fe_{2}B}=3.71\cdot 10^{-4}exp\left(\frac{-193.47\frac{\mathrm{kJ}}{\mathrm{mol}}}{RT}\right)\;\;\;m^{2}/s$$

where *R* represents the ideal gas constant (8.314 J/mol. K) and *T* represents the process temperature expressed in K.

Table 5 contains the boron activation energies obtained in the literature for some steels treated by various boronizing processes along with the results found in the current work. The reported values in the literature are dependent on different factors, including differences in the chemical composition of the steels, the calculation method for the boron activation energies, differences in boronizing parameters (including duration and temperature), the boronizing method employed, and the nature of the chemical or electrochemical reactions occurring in this thermochemical process. To highlight the differences regarding the obtained activation energies from the literature, possible explanations are given hereafter. For example, Keddam and Topuz [27] used the powder method via indirect heating in a fluidized bed to treat the 34CrAlNi7 steel between 1123 and 1323 K. The activation energies of boron in FeB and Fe_2_B were determined using the integral method [27]. The assessed activation energies were lower to those in the present work due to the difference in chromium content between the 34CrAlNi7 and X165CrV12 steels and the chemical composition of the boriding agents. In ref. [33], the integral method and Dybkov model were used to deduce the activation energies of boron in FeB and Fe_2_B for AISI M2 steel. Accordingly, the assessed activation energies of boron were higher, resulting from the direct influence of the alloying elements of AISI M2 steel on boron mobility, independent of the used model. The comparable values of boron activation energies were calculated for powder-pack borided AISI D2 steel using mean diffusion coefficient method (MDC) [34]. The significantly higher values were characteristic of borided ASP®2012 steel [35]. In a recent work, Turkoglu and Ay [36] compared the pack-boriding kinetics of AISI 304, AISI 420, and AISI 430 steels in the interval of 1123–1273 K. The deduced boron activation energies for these three steels were quite different and contradicted the literature results to some extent. For instance, the obtained activation energy (151.373 kJmol^−1^) for AISI 430 steel (containing 16.9 wt.%Cr and 0.09 wt.%Ni) was much lower than that (242.153 kJmol^−1^) of AISI 420 steel (13.3 wt.% Cr and 0.09 wt.% Ni) in spite of the increased chromium content by a factor of 1.27 and equivalent nickel content.

Campos-Silva [37] utilized a recent boriding process, named the pulsed-direct current powdered method, to surface harden the substrates of AISI 316 L steel. In this process, boron diffusion was enhanced under the action of an electrical field and with the possibility of changing polarity for a constant input current. A bilayer model was suggested to deduce the values of boron activation energies in FeB and Fe_2_B. The estimated activation energies were diminished compared to those of conventional powder-pack boriding, due to effect of the electrical field that promoted the diffusion of boron atoms by producing thicker layers for shorter times (less or equal to 2 h). Keddam et al. [38] boronized AISI 316 steel by plasma-paste boriding with 100% B_2_O_3_ in the temperature interval of 970–1073 K. The estimated boron activation energy for this steel was 118.12 kJ mol^−1^, which was the lowest value of those [27,33,34,35,36,37,38,39,40] listed in Table 5 owing to the activation of plasma energy. This situation brought about lowering of the boron activation energy of the treated AISI 316 steel. In other research work, Arslan et al. [39] used a new thermochemical process, called CRTD-Bor (cathodic reduction and thermal diffusion-based boriding), for boronizing AISI 314 L steel between 950 and 1050 °C. The boriding medium was composed of 90 wt.% Na_2_B_4_O_7_ and 10% Na_2_CO_3_ under a constant current density of 200 mA cm^−2^ for 0.25 to 1 h. The key benefit of a such process was the generation of thick boride layers in shorter times. Furthermore, the determined boron activation was 181.45 kJ mol^−1^ for AISI 314 L steel. Sen et al. [40] used a salt bath to surface harden AISI D2 steel in the range of 1073 to 1273 K for 2, 4, 6, and 8 h, thus forming the dual boride layer (FeB + Fe_2_B). The slurry salt bath method was used for this purpose with a reaction medium containing boric acid, borax, and ferro-silicon. The calculated boron activation energy with the classical parabolic growth law was 170 kJ mol^−1^ with the entire layer thickness ranging from 20 to 88 µm. In addition, nonlinear fittings with different equations (Paraboloid, Gaussian, and Lorentzian) were proposed to predict the entire layer thickness with changes in the boriding parameters.

It is evident that the activation energies of boron derived from the present work are in line with other results [27,33,34] displayed in Table 5 for powder-pack boriding. However, the boron activation energy reported by Kayali et al. [35] (314.716 kJ mol^−1^) for ASP^®^2012 steel was the highest among other values (Table 5). It should be emphasized that the boron activation energy determined in the present work for the FeB phase (173.73 kJ mol^−1^) was lower than that for the Fe_2_B phase (193.47 kJ mol^−1^). This suggested that the FeB phase could be formed before the Fe_2_B phase appeared in the microstructure, which would be consistent with previous observations of gas boriding [1].

The limitations resulting from the application of different approaches [27,33,34,37] for describing the boronizing kinetics of steels should be highlighted. In fact, in the case of alloyed steels, the contribution of boron atoms needed for the precipitation of metal borides was ignored during boron diffusion in which the mutual carbon-boron interaction was disregarded. In spite of these limitations, the present integral diffusion model remains applicable for the boronizing kinetics of X165CrV12 steel.

### 3.3. Microhardness Profiles

**Figure 14:**
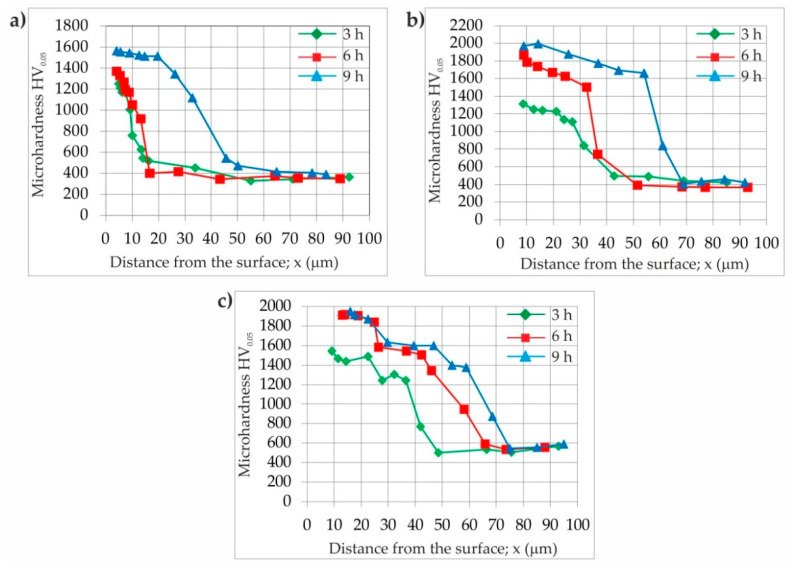
Microhardness profiles of X165CrV12 steel after powder-pack boriding at different temperature: (**a**) 1123 K, (**b**) 1173 K and (**c**) 1223 K.

The Vickers microhardness profiles versus the distance from the surface are presented in Figure 14. For all of the analyzed samples, the highest hardness value was measured in the FeB and Fe_2_B zones. However, some differences were visible when comparing the profiles obtained at the same temperature and different boriding times. In general, the increase in boriding duration resulted in an increase in the hardness of the iron borides. This situation could be caused by the higher degree of substitution of the iron atoms in MeB and Me_2_B borides by chromium atoms. The boriding temperature had a similar effect on the hardness measured. In the case of high-carbon tool steel, the hardness of the borided layers depended mainly on the temperature and time of boriding, as well as on the alloying elements present in the substrate material. Despite the high content of carbon in X165CrV12 steel, this element had a negligible influence on the hardness of the borided layer due to the insolubility of carbon in the iron borides [8,41]. The decreased hardness was measured in the transition region below the iron borides layer (Figure 14). However, the presence of a high amount of alloyed carbides with high chromium content caused the increased hardness in this region compared to that in the substrate material.

### 3.4. Nanomechanical Properties

**Figure 15:**
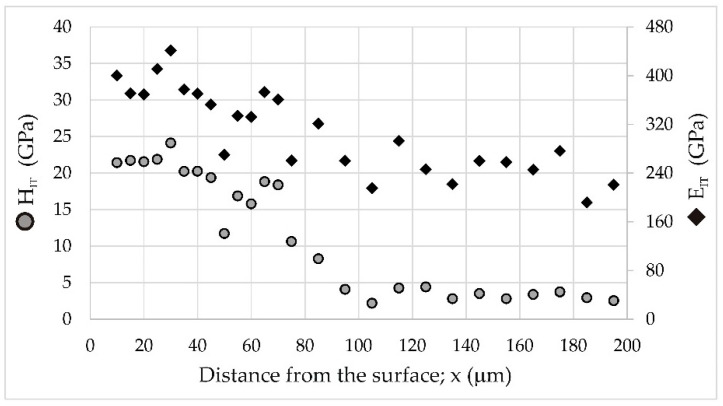
Indentation hardness (*H_IT_*) and indentation Young’s modulus (*E_IT_*) profiles of X165CrV12 steel after powder-pack boriding at the temperature of 1223 K for 9 h.

**Figure 16:**
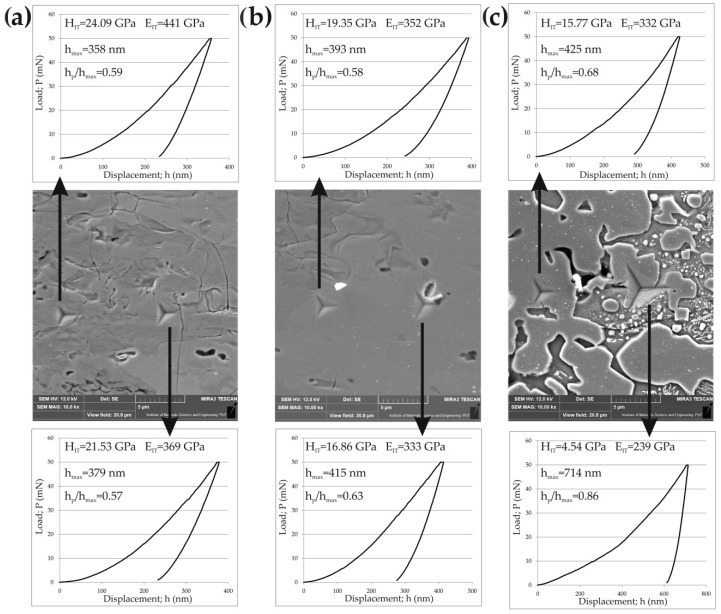
SEM images of X165CrV12 steel after powder-pack boriding at the temperature of 1223 K for 9 h with visible selected indents and the results of nanoindentation performed in: (**a**) outer FeB zone, (**b**) inner Fe_2_B zone and (**c**) transition zone.

Although CrB borides were identified in the outer FeB zone and Cr_2_B borides in the Fe_2_B zone by XRD, their amount was relatively small. Additionally, chromium could replace iron in complex borides (Fe,Cr)B or (Fe,Cr)_2_B. Hence, it would be difficult to measure the hardness and Young’s modulus separately for the iron and chromium borides. Therefore, the measured nanomechanical properties were characteristic of the entire FeB and Fe_2_B zones. Figure 15 illustrates the indentation hardness (*H_IT_*) and indentation Young’s modulus (*E_IT_*) profiles vs. the distance from the surface. As expected, the highest indentation hardness (19.35–24.09 GPa) was measured in the FeB zone. The average hardness of this zone was 21.88 ± 1.35 GPa, which was higher than the hardness measured in the FeB phase (20.95 ± 0.93 GPa) produced on the Armco iron substrate by gas boriding [4]. This situation was caused by the presence of CrB borides in the outer FeB zone. A similar effect was observed in the case of the borided layer formed on 304 stainless steel [18]. Its properties after boriding were compared with those characteristic of the layer produced on H13 hot work tool steel. The increased concentration of chromium in 304 stainless steel (18.3 wt%) was the reason for higher hardness of the borided layer compared to the layer formed on H13 steel (5.3 wt.% Cr). This was caused by a higher percentage of chromium borides in the borided layer produced on the steel substrate with a higher chromium content [18]. This could also prove the existence of (Fe,Cr)B and (Fe,Cr)_2_B phases. It was also proven that even a small increase in the concentration of alloying elements in steel, including chromium, resulted in an increase in the hardness of the boride layer produced [42]. Therefore, the oscillations of the indentation hardness were visible, as shown in Figure 15. A similar tendency was observed for the Fe_2_B zone characterized by an average hardness of 17.45 ± 1.20 GPa, which was lower than that of the outer FeB zone. The effect of the distance from the surface on Young’s modulus was also observed (Figure 15). A higher indentation Young’s modulus was obtained in the outer FeB zone (386.27 ± 27.04 GPa) than in the inner Fe_2_B zone (339.75 ± 17.44 GPa). The transition zone (at the depth between 75 and 95 µm from the top surface) was characterized by an interesting profile of *H_IT_* and *E_IT_*. The differences between the lowest and highest values were significant (Figure 15). This situation resulted from the accumulation of chromium atoms below the iron borides zone (Figure 8, Figure 9 and Figure 10). To explain these differences, a detailed analysis of the location of selected Berkovich indents in the microstructure of the borided X165CrV12 steel (Figure 16) was useful.

Figure 16 presents SEM images of borided X165CrV12 steel with visible Berkovich indents performed in the outer FeB zone (Figure 16a), inner Fe_2_B zone (Figure 16b), and the transition zone (Figure 16c). The smallest Berkovich indent dimensions were characteristic of the FeB zone (Figure 16a). However, in the area closer to the top surface, higher indentation hardness and indentation Young’s modulus were obtained, at 24.09 GPa and 441 GPa, respectively. The second indent performed in this zone had a lower *H_IT_* of 21.53 GPa, as well as a lower *E_IT_* of 369 GPa. These differences were a direct result of the chromium content measured in these areas (Figure 8). In the area in which the concentration of chromium was higher, and thus the amount of CrB borides was increased, higher values of nanomechanical properties were measured, including hardness (*H_IT_*), Young’s modulus (*E_IT_*), and maximum penetration depth (*h_max_*). A similar tendency was observed in the case of the Berkovich indents performed in the Fe_2_B zone (Figure 16b). The nanomechanical properties measured in the transition zone strongly depended on the location of the indent (Figure 16c). The direct interface between the Fe_2_B needle tips and the substrate material was characterized by an increased chromium concentration (Figure 8). Therefore, the Berkovich indent performed in this region was characterized by a high hardness of 15.77 GPa and high Young’s modulus of 332 GPa. When the indent was made in the substrate with precipitates of alloyed carbides, the predominant influence of the matrix on the results was visible. The measured hardness and Young’s modulus were significantly lower, at 4.54 GPa and 239 GPa, respectively. Iron borides represent a group of phases with high hardness, high elastic modulus, and low maximum penetration depth, in contrast to the substrate with carbides. Comparing the shape of the load displacement curves, as well as the ratio of the permanent penetration depth *h_p_* to the maximum penetration depth *h_max_*, it was found that both iron boride zones demonstrated more elastic behavior than the substrate with carbides.

### 3.5. Wear Resistance

**Figure 17:**
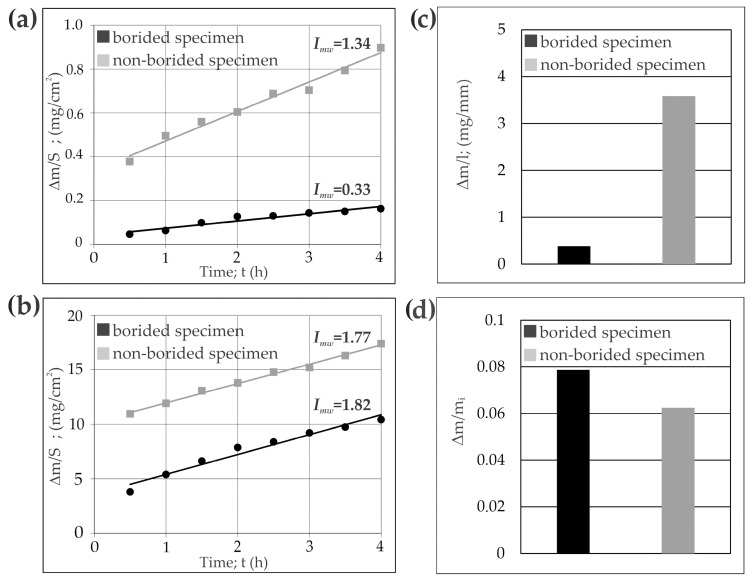
Results of wear resistance tests: (**a**) mass loss of specimens on a unit of friction surface vs. time of friction with calculated *I_mw_* factors, (**b**) mass loss of counter-specimens on a unit of friction surface vs. time of friction with calculated *I_mw_* factors, (**c**) ratio of mass loss of specimens to length of wear path, (d) relative mass loss of counter-specimens.

The tribological properties of the borided layer produced at 1223 K for 9 h were investigated. This layer was characterized by the highest thickness. In general, the borided layers produced on X165CrV12 steel had a relatively small thickness due to high concentrations of carbon and chromium. The aim of this study was to determine whether the thickest of these layers could be used under heavy load conditions. The results were compared to those obtained for non-borided X165CrV12 steel. Three parameters describing the wear resistance of specimens and counter-specimens were determined: the mass wear intensity factor (*I_mw_*) (Figure 17a,b) of both specimens and counter-specimens, the ratio of specimens’ mass loss to the length of wear path (∆*m*/*l*) (Figure 17c), and the relative mass loss of the counter-specimens (∆*m/m_i_*) (Figure 17d). The formation of a hard borided layer on X165CrV12 steel provided higher wear resistance, as expressed by the mass wear intensity factor (Figure 17a). The borided sample was characterized by a four time lower value of *I_mw_* (0.33 mg/cm^2^) compared to the non-borided X165CrV12 steel (*I_mw_* = 1.34 mg/cm^2^). However, the *I_mw_* coefficient determined for the counter-samples was lower for a friction pair consisting of a non-borided specimen and a quenched and low-temperature-tempered 100CrMnSi6-4 bearing steel as a counter-specimen. This result also showed the increased wear resistance of the powder-pack borided layer. The values of the ratio of mass loss to the length of wear path measured for the specimens indicated the significantly diminished ∆*m*/*l* value for the borided specimen (nine times lower) compared to that of the non-borided material. The values of the relative mass loss calculated for the counter-specimens (Figure 17d) confirmed that the boriding process was an effective method of improving the wear resistance of tool steels. The diminished ∆*m/m_i_* ratio (0.06251) was characteristic of a counter-specimen mating with the non-borided sample compared the counter-specimen mating with the borided material (0.07866).

## 4. Summary and Conclusions

The powder-pack boriding method with an open retort was used to produce borided layers on X165CrV12 tool steel. Different process parameters were used, including temperatures of 1123, 1173, and 1223 K and boriding times of 3, 6, and 9 h. The growth kinetics and some mechanical properties of the borided X165CrV12 steel were investigated. Based on the detailed analysis of the obtained results, the following conclusions were formulated:

- The total thickness of the produced layers strongly depended on the boriding parameters. The increased temperature and longer duration were accompanied by an increase in the thickness of the borided layer.
- The produced layers were composed of three zones: the outer FeB layer (1), the inner Fe_2_B boride zone (2) and the transition zone (3), below which the substrate material (4) was observed.
- As a consequence of the high concentration of chromium in X165CrV12 steel, the borided layers also contained CrB borides.
- The transition zone was characterized by increased chromium content, and its microstructure was composed of fine pearlite, secondary carbides, and ledeburite—a transformed eutectic mixture—which included fine pearlite and primary and secondary alloyed carbides. This was related to the accumulation of chromium and carbon atoms at the interface between tips of Fe_2_B needles and the substrate material.
- The integral method was utilized to describe the growth kinetics of the FeB and (FeB + Fe_2_B) layers on the surface of powder-pack borided X165CrV12 tool steel. In general, the boron activation energies derived from the present work were in line with reported results for powder-pack boriding. It was important that the boron activation energy, determined in the present work for the FeB phase, was lower than that for the Fe_2_B phase. This confirmed the previous observations after gas boriding and suggested that the FeB phase could be formed before the Fe_2_B phase appeared in the microstructure.
- In the case of high-carbon tool steel, the hardness of the borided layers depended mainly on the temperature and time of boriding, as well as on the alloying elements present in the substrate material. Despite the high content of carbon in X165CrV12 steel, this element had a negligible influence on the hardness of the borided layer due to the insolubility of carbon in the iron borides. In general, the increase in boriding temperature and time resulted in increased hardness of the borided layer. This was a consequence of the higher amount of hard chromium borides in the microstructure.
- The nanomechanical properties of the borided layer produced on X165CrV12 steel strongly depended on the selected testing area. The highest indentation hardness (*H_IT_* = 21.88 ± 1.35 GPa) and indentation Young’s modulus (*E_IT_* = 386.27 ± 27.04 GPa) were measured in the FeB zone. This situation was caused by the presence of CrB borides in the outer FeB zone.
- In the transition zone, as a result of the accumulation of chromium atoms below the iron borides zone, high hardness (15.77 GPa) and Young’s modulus (332 GPa) were obtained in some areas. This was related to the presence of alloyed carbides in this region.
- The wear tests showed improved wear resistance of the borided X165CrV12 tool steel in comparison to the non-borided specimen. The mass wear intensity factor (*I_mw_*) was four times lower for the borided material. Simultaneously, the borided X165CrV12 steel had a nine times lower ratio of mass loss to the length of wear path (*Δm/l*) in comparison to the non-borided X165CrV12 steel.

## Figures and Tables

**Table 1:** Chemical composition of X165CrV12 tool steel used as a substrate material.

Element	C	Cr	Mn	Si	V	P	S
wt.%	1.55–1.75	11.00–12.00	0.20–0.40	0.25–0.40	0.07–0.12	≤0.035	≤0.035

**Table 2:** The results of EDS X-ray microanalysis performed in areas marked in Figure 6.

Element Concentration (wt.%)	Area 1	Area 2	Area 3	Area 4	Area 5	Area 6	Area 7
Fe	47.5	74.2	73.0	77.2	82.7	83.6	88.4
Cr	33.7	7.7	10.7	13.6	9.0	15.3	11.6
B	18.8	18.1	16.3	9.2	8.3	1.1	0.0

**Table 3:** Experimental parabolic growth constants deduced from the data of Figure 12.

Temperature (K)	*k’* (µm s^−0.5^) at the First Interface	*k* (µm s^−0.5^) at the Second Interface
1123	0.088	0.1839
1173	0.1486	0.3142
1223	0.1934	0.4262

**Table 4:** Assessed boron diffusion coefficients in in iron borides with Equations (24) and (25).

Temperature (K)	$D_{FeB}$ (×10^−12^ m^2^ s^−1^) Equation (24)	$D_{Fe_{2}B}$ (×10^−12^ m^2^ s^−1^) Equation (25)	ε Parameter	*η* Parameter
1123	0.41	0.35	0.0689	0.1556
1173	1.17	1.03	0.0687	0.1551
1223	2.02	1.89	0.0681	0.1537

**Table 5:** Comparison of reported activation energies with the present results.

Steel	Boronizing Treatment	Interval of Temperature (K)	Activation Energy (kJ mol^−1^)	Approach Utilized	Refs.
34CrAlNi7	Powder-pack	1123–1323 for 1–4 h	169.4 (FeB) 165.27 (Fe_2_B)	Integral method	[27]
AISI M2	Powder-pack	1173–1323 For 4–10 h	206.41(FeB) 216.18 (Fe_2_B)	Integral method	[33]
AISI M2	Powder-pack	1173–1323 For 4–10 h	226.02 (FeB) 209.04 (Fe_2_B)	Dybkov model	[33]
AISI D2	Powder-pack	1223–1273 For 3–10 h	208.04 (FeB) 197.46 (Fe_2_B)	MDC method	[34]
ASP^®^2012	Powder-pack	1123–1223 For 2–6 h	314.716	Parabolic relationship	[35]
AISI 304	Powder-pack	1123–1273 For 2–6 h	182.359	Parabolic relationship	[36]
AISI 420	Powder-pack	1123–1273 For 2–6 h	242.153	Parabolic relationship	[36]
AISI 430	Powder-pack	1123–1273 For 2–6 h	151.373	Parabolic relationship	[36]
AISI 316 L	Pulsed DC powder	1123–1273 For 0.5–2 h	162 (FeB) 171 (Fe_2_B)	Diffusion model	[37]
AISI 316	Plasma-paste boriding (PPB)	973–1073 For 3–7 h	118.12	Classical parabolic growth law	[38]
AISI 304	CRTD-Bor	1223–1323 For 0.25–1 h	181.46	Classical parabolic growth law	[39]
AISI D2	Salt bath	1073–1273 For 2–8 h	170	Classical parabolic growth law	[40]
X165CrV12	Powder-pack with an open retort	1123–1223 For 3–9 h	173.73 (FeB) 193.47 (Fe_2_B)	Integral diffusion model	Present work

## Data Availability

The authors confirm that the data supporting the findings of this study are available within the article.
